# A Case–Control Study of *ADCY9* Gene Polymorphisms and the Risk of Hepatocellular Carcinoma in the Chinese Han Population

**DOI:** 10.3389/fonc.2020.01450

**Published:** 2020-08-25

**Authors:** Xu Chao, Yong Jia, Xuesong Feng, Guoquan Wang, Xiaoping Wang, Hailong Shi, Fei Zhao, Chao Jiang

**Affiliations:** ^1^The College of Basic Medicine, Shaanxi University of Chinese Medicine, Xianyang, China; ^2^The Second Affiliated Hospital, Shaanxi University of Chinese Medicine, Xianyang, China; ^3^The Affiliated Hospital, Shaanxi University of Chinese Medicine, Xianyang, China; ^4^The College of Pharmacy, Shaanxi University of Chinese Medicine, Xianyang, China; ^5^Department of Neurology, The Second Affiliated Hospital of Xi'an Medical University, Xi'an, China

**Keywords:** *ADCY9*, polymorphisms, hepatocellular carcinoma risk, stratification analysis, case–control study

## Abstract

**Background:** Adenylyl cyclase type 9 (*ADCY9*) modulates signal transduction by producing the second messenger cyclic AMP. It has been reported that *ADCY9* gene polymorphisms were associated with cancer development. The aim of this study was to investigate whether *ADCY9* gene polymorphisms could contribute to the susceptibility of hepatocellular carcinoma (HCC) in the Chinese Han population.

**Methods:** In the present study, five single-nucleotide polymorphisms (SNPs) in *ADCY9* were genotyped using Agena MassARRAY platform in 876 subjects from China. Logistic regression was used to assess the effects of SNPs on HCC risk. Associations were also evaluated for HCC risk stratified by age and gender. False discovery rate (FDR) was used to correct multiple testing.

**Results:** After adjusting for age and gender, we found a significant relationship between heterozygous genotypes of rs2531995 and HCC risk (OR = 1.34, 95% CI = 1.01–1.77, *p* = 0.045). *ADCY9* rs2230742 had a strong relationship with lower risk of HCC in allele (*p* = 0.006), co-dominant (*p* = 0.023), dominant (*p* = 0.010), and additive (*p* = 0.006) models. Stratified analysis showed that rs879620 increased HCC risk and rs2230742 was associated with lower risk of HCC in the individuals aged 55 or younger, rs2531992 significantly decreased HCC risk in the elder group (age > 55). For women, rs2230742 and rs2230741 were significantly associated with HCC risk in multiple models (*p* < 0.05). FDR analysis showed that rs2230742 could protect individuals from HCC risk in the allele model (FDR*-p* = 0.030). In addition, haplotype analysis indicated that C_rs879620_A_rs2230742_A_rs2230741_ haplotype was a protective factor for HCC (OR = 0.67, 95% CI = 0.50–0.89, *p* = 0.007, FDR*-p* = 0.028).

**Conclusion:** Our findings suggest that *ADCY9* gene polymorphisms are associated with HCC risk in the Chinese Han population.

## Introduction

Hepatocellular carcinoma (HCC) is the most common malignant tumor in the liver (1). Although mechanism of hepatocellular carcinogenesis is still not fully clear, it is widely recognized that HCC development is the consequence of complex interactions between the genome, lifestyle, and environment (2). Genome-wide association studies have highlighted that genetic variants might play a vital role in HCC susceptibility (3). It is reported that HCC is the fifth most common cancer and the third leading cause of cancer mortality worldwide (4). The prevalence of HCC is the highest in East, Southeast Asia, and Sub-Saharan Africa, especially in China, which accounts for ~50% of all cases (5, 6). Moreover, the prevalence of HCC is increasing with age and in men (7, 8). Hence, understanding the effects of genetic variants in different populations is highly important for studying the mechanisms of HCC and applying these results to risk prediction.

The adenylyl cyclase type 9 (*ADCY9*) gene belongs to the adenylyl cyclase (AC) gene family, which produces the second messenger cyclic AMP (adenosine-3′,5′-monophosphate) in response to G protein-coupled receptors (GPCRs) activation and codes for the protein AC type 9, an integral membrane protein composed of 12 transmembrane segments (9, 10). Previous studies have shown that *ADCY9* gene polymorphisms were related to the development of many diseases, including cardiovascular diseases, mood disorders, malaria, asthma, and allergy (2, 10–12). The potential function of *ADCY9* in cancer development was also reported. Yongzhen et al. found that *ADCY9* mutation altered bladder cancer development (13). The involvement of *ADCY9* in phospholipase C signaling has effects on colorectal cancer progression and metastasis (14). Elevated expression of *ADCY9* is a potential prognostic biomarker for patients with colon cancer (9). Nonetheless, there were no studies that focused on the relationship between *ADCY9* gene polymorphisms and HCC risk.

Hence, we hypothesized that *ADCY9* gene polymorphisms could play an important role in the progression of HCC. This case–control study was conducted to assess the effects of five single-nucleotide polymorphisms (SNPs) in *ADCY9* gene on the risk of HCC among the Chinese Han population. To the best of our knowledge, this is the first study to investigate the impact of *ADCY9* SNPs for HCC susceptibility in the Chinese Han population.

## Materials and Methods

### Study Subjects

A total of 434 HCC patients and 442 age- and gender-matched healthy individuals were enrolled in this study. The patients were diagnosed as HCC by histology or pathology in the hospital, and the controls were healthy individuals without family history diseases, cardiovascular diseases, autoimmune diseases, respiratory diseases, cancers, or other severe diseases derived randomly from the same hospital. None of the patients had received radiation, chemotherapy, or surgical therapy before joining our study. Characteristic information was obtained from their medical records, including age, gender, smoking, and drinking status.

### DNA Extraction and Genotyping

Peripheral blood samples were collected from the participants and were stored at −80°C until analysis. Genomic DNA was extracted from EDTA-containing blood via a blood DNA kit (GoldMag Co. Ltd., Xi'an, China). DNA concentration was measured by Nanodrop 2000 (Thermo Scientific, Waltham, Massachusetts, USA) (15). Combined with previous studies and the criteria of minor allele frequency (MAF) ≥ 0.05, five SNPs (rs2531995, rs879620, rs2230742, rs2230741, and rs2531992) were selected in this study based on the dbSNP database (https://www.ncbi.nlm.nih.gov/snp/) and HapMap database (http://www.hapmap.org). Primers were designed by MassARRAY Assay Design 3.0 software and were listed in Supplemental Table 1. SNP genotyping was performed by the MassARRAY iPLEX platform (Agena Bioscience, San Diego, CA, USA) (16). Finally, the data analysis was accomplished by Agena Bioscience TYPER version 4.0 software (17).

### Genetic Models

For most SNPs, they often contain two types of alleles: a minor allele “A” with low frequency and a wild allele “B” with high frequency. Four genetic models were identified based on the alleles: co-dominant model BB vs. AB vs. AA, dominant BB vs. AB+AA, recessive BB+AB vs. AA, and log-additive: for each A increase. These models were applied to further detect the risk effect of minor allele on disease in a population. In the manuscript, we defined the allele with low frequency as the minor allele “A,” and the other was the wild allele “B.” Furthermore, four genetic models (codominant: BB vs. AB vs. AA, dominant: BB vs. AB+AA, recessive: BB+AB vs. AA, and log-additive: for each A increase) were employed using SNPstats software (https://www.snpstats.net/start.htm) to estimate the relationship between each SNP and EC risk.

### Statistical Analysis

The statistical analyses were performed using the SPSS 17.0 (IBM®, Armonk, New York, USA). The Hardy–Weinberg equilibrium (HWE) in the healthy control was assessed by Fisher exact test. A chi-square test was performed to compare the categorical variable—sex. We used *t-*test to assess the difference in age. Association analysis based on logistic regression was performed by estimating odds ratios (OR) and 95% confidence intervals (CI) with multiple models for each SNP. We used Power and Sample Size Calculation software (http://sampsize.sourceforge.net/iface/s3.html#ccp) to calculate the power of the significant difference. False discovery rate (FDR) was used to correct multiple testing. The haplotype analysis and linkage disequilibrium (LD) were conducted by PLINK software and Haploview software (version 4.2) (18). *p* < 0.05 was considered statistically significant in all tests. In addition, HaploReg v4.1 (https://pubs.broadinstitute.org/mammals/haploreg/haploreg.php) was used to predict the possible functional effects on the selected SNPs.

## Results

### Characteristics of the Participants

The characteristics of the 876 subjects (434 cases and 442 controls) are shown in Table 1. There was no significant difference in age (cases: 55.10 ± 10.19, controls: 55.16 ± 11.54; *p* = 0.941) and gender (cases: 80% men; controls: 80% men) between two groups. In addition, smoking and drinking status of all participants are presented in Table 1.

**Table 1:** Characteristics of hepatocellular carcinoma patients and healthy subjects.

**Variable**	**HCC group (*N* = 434)**	**Healthy group (*N* = 442)**	***p***
Age (mean ± SD)	55.10 ± 10.19	55.16 ± 11.54	0.941
>55	211 (49%)	192 (43%)	
≤55	223 (51%)	250 (57%)	
Sex			0.867
Men	347 (80%)	351 (80%)	
Women	87 (20%)	91 (20%)	
Smoking			
Yes	81 (19%)	152 (34%)	
No	62 (14%)	129 (29%)	
Absence	291 (67%)	161 (36%)	
Drinking			
Yes	55 (13%)	133 (30%)	
No	79 (18%)	118 (27%)	
Absence	300 (69%)	191 (43%)	

*HCC, Hepatocellular carcinoma*.

*“Absence” means no data or unclear*.

### The Basic Information and Potential Function of the Selected SNPs

As shown in Table 2, the genotypic frequencies of the *ADCY9* gene polymorphisms among the controls were in accord with HWE (*p* > 0.05) and the MAF of *ADCY9* gene polymorphisms were larger than 0.05. In allelic tests, we found *ADCY9* rs2230742 significantly decreased the risk of HCC (OR = 0.67, 95% CI = 0.50–0.89, *p* = 0.006, FDR-*p* = 0.030). However, there was no significant association between other SNPs and risk of HCC. Besides, these selected SNPs had association with “Promoter histone marks,” “Enhancer histone mark,” “SiPhy cons,” “DNAse,” “Motifs changed,” “NHGRI/EBI GWAS hits,” “GRASP QTL hits,” and “Selected eQTL hits” by online tool.

**Table 2:** Summarized information of selected SNPs in the *ADCY9* gene.

**SNPs**	**bp position**	**Aliases**	**Minor/Major alleles**	**MAF-case**	**MAF-control**	**HWE *p***	**OR (95% CI)**	***p***	**FDR-*p***	**HaploReg**
rs2531995	Chr16: rs253195	157720G>A	T/C	0.148	0.146	0.152	1.17 (0.96–1.43)	0.120	0.200	Promoter histone marks; Enhancer histone marks; DNAse; NHGRI/EBI GWAS hits; GRASP QTL hits; Selected eQTL hits
rs879620	Chr16: 3965728	155458G>A	T/C	0.351	0.316	0.438	1.19 (0.97–1.45)	0.090	0.225	DNAse; Motifs changed; GRASP QTL hits; Selected eQTL hits
rs2230742	Chr16: 3966675	154511T>G	A/G	0.349	0.311	0.848	0.67 (0.50–0.89)	**0.006**	**0.030**	SiPhy cons; DNAse; Motifs changed
rs2230741	Chr16: 3966942	154244T>C	G/A	0.103	0.146	0.775	1.13 (0.90–1.41)	0.301	0.301	SiPhy cons; Enhancer histone marks; Motifs changed
rs2531992	Chr16: 3971733	149453T>C	A/G	0.232	0.212	0.513	0.81 (0.60–1.09)	0.170	0.213	Promoter histone marks; Enhancer histone marks; DNAse; Motifs changed

*SNP, Single-nucleotide polymorphism; MAF, Minor allele frequency; HWE, Hardy–Weinberg equilibrium; OR, Odds ratios; CI, Confidence intervals; FDR, False discovery rate*.

*Significant values are marked in bold*.

### Association Between *ADCY9* Gene Polymorphisms and Risk of HCC

Among the five SNPs in *ADCY9* gene, rs2531995 and rs2230742 were significantly associated with HCC risk (Table 3). In contrast to homozygous wild-type alleles, heterozygote T/C of rs2531995 remarkably increased the risk of HCC (adjusted OR = 1.34, 95% CI = 1.01–1.77, *p* = 0.045). rs2230742 was identified to decrease the HCC risk in codominant (adjusted OR = 0.68, 95% CI = 0.49–0.95, *p* = 0.023), dominant (adjusted OR = 0.66, 95% CI = 0.48–0.90, *p* = 0.010), and additive (adjusted OR = 0.67, 95% CI = 0.50–0.89, *p* = 0.006) models. The other three SNPs showed no significant evidence of an association with HCC.

**Table 3:** Associations between *ADCY9* gene polymorphisms and hepatocellular carcinoma risk.

**SNP**	**Model**	**Genotype**	**Without adjustment**		**With adjustment**		**Study Power**	**FDR-*p***
			**OR (95% CI)**	***p***	**OR (95% CI)**	***p***		
rs2531995	Codominant	TT	1.20 (0.78–1.85)	0.411	1.21 (0.78–1.87)	0.401		0.477
T < C		TC	1.34 (1.01–1.77)	**0.045**	1.34 (1.01–1.77)	**0.045**	100%	0.281
		CC	1.00		1.00			
	Dominant	TT-TC	1.31 (1.00–1.71)	0.050	1.31 (1.00–1.71)	0.050		0.250
		CC	1.00		1.00			
	Recessive	TT	1.04 (0.69–1.57)	0.848	1.05 (0.69–1.58)	0.831		0.903
		TC-CC	1.00		1.00			
	Additive		1.17 (0.96–1.42)	0.126	1.17 (0.96–1.42)	0.121		0.303
rs879620	Codominant	TT	1.30 (0.83–2.03)	0.251	1.30 (0.83–2.04)	0.247		0.363
T < C		TC	1.29 (0.97–1.71)	0.080	1.29 (0.97–1.70)	0.082		0.293
		CC	1.00		1.00			
	Dominant	TT-TC	1.29 (0.99–1.68)	0.063	1.29 (0.99–1.68)	0.063		0.263
		CC	1.00		1.00			
	Recessive	TT	1.15 (0.75–1.75)	0.526	1.15 (0.75–1.76)	0.513		0.583
		TC-CC	1.00		1.00			
	Additive		1.19 (0.97–1.45)	0.093	1.19 (0.97–1.45)	0.091		0.284
rs2230742	Codominant	AA	0.37 (0.11–1.19)	0.095	0.37 (0.11–1.19)	0.094		0.261
A < G		AG	0.69 (0.50–0.95)	**0.023**	0.68 (0.49–0.95)	**0.023**	100%	0.192
		GG	1.00		1.00			
	Dominant	AA-AG	0.66 (0.48–0.90)	**0.010**	0.66 (0.48–0.90)	**0.010**	100%	0.125
		GG	1.00		1.00			
	Recessive	AA	0.40 (0.12–1.29)	0.125	0.40 (0.12–1.29)	0.125		0.284
		AG-GG	1.00		1.00			
	Additive		0.67 (0.50–0.89)	**0.006**	0.67 (0.50–0.89)	**0.006**	100%	0.150
rs2230741	Codominant	GG	1.04 (0.55–1.97)	0.897	1.04 (0.55–1.97)	0.902		0.940
G < A		GA	1.22 (0.92–1.61)	0.174	1.22 (0.92–1.61)	0.174		0.335
		AA	1.00		1.00			
	Dominant	GG-GA	1.19 (0.91–1.57)	0.200	1.19 (0.91–1.57)	0.200		0.357
		AA	1.00		1.00			
	Recessive	GG	0.97 (0.52–1.82)	0.926	0.97 (0.52–1.81)	0.920		0.920
		GA-AA	1.00		1.00			
	Additive		1.13 (0.90–1.42)	0.298	1.13 (0.90–1.42)	0.300		0.395
rs2531992	Codominant	AA	1.04 (0.55–1.97)	0.897	0.48 (0.14–1.62)	0.238		0.372
A < G		AG	1.22 (0.92–1.61)	0.174	0.85 (0.61–1.19)	0.338		0.423
		GG	1.00		1.00			
	Dominant	AA-AG	0.82 (0.59–1.14)	0.238	0.82 (0.59–1.14)	0.234		0.390
		GG	1.00		1.00			
	Recessive	AA	0.50 (0.15–1.69)	0.267	0.50 (0.15–1.67)	0.260		0.361
		AG-GG	1.00		1.00			
	Additive		0.81 (0.61–1.09)	0.173	0.81 (0.6–1.09)	0.168		0.350

*SNP, Single-nucleotide polymorphism; OR, Odds ratios; CI, Confidence intervals; FDR, False discovery rate*.

*Significant values are marked in bold*.

*With adjustment means adjusted by age and gender*.

### Stratified Analysis Based on Age and Gender

To further assess the five potential susceptible polymorphisms to the risk of HCC, a stratified analysis was performed by subgroups of participants' age and gender (Tables 4, 5). In the subgroup of age > 55, rs2531992 was associated with decreased HCC risk in additive (adjusted OR = 0.64, 95% CI = 0.42–0.99, *p* = 0.044) and allele (adjusted OR = 0.64, 95% CI = 0.42–0.99, *p* = 0.044) models. Furthermore, in the group of age ≤ 55, rs879620 was associated with higher risk of HCC in multiple models (dominant: adjusted OR = 1.50, 95% CI = 1.04–2.18, *p* =0.031; additive: adjusted OR = 1.35, 95% CI = 1.04–1.76, *p* = 0.026; allele: adjusted OR = 1.35, 95% CI =1.03–1.76, *p* = 0.028), whereas rs2230742 was related to lower risk of HCC. For the subgroup of gender, rs2230742 also significantly decreased HCC risk in the women group (dominant: adjusted OR = 0.43, 95% CI = 0.21–0.88, *p* =0.022; additive: adjusted OR = 0.42, 95% CI = 0.21–0.83, *p* = 0.012; allele: adjusted OR = 0.43, 95% CI = 0.22–0.83, *p* = 0.011). The rs2230741 polymorphism showed an increased relationship with HCC risk in dominant (adjusted OR = 1.91, 95% CI = 1.04–3.50, *p* = 0.037) and additive (adjusted OR = 1.78, 95% CI = 1.04–3.06, *p* = 0.037) models, and the minor allele G of rs2230741 was also related to increasing HCC risk (adjusted OR = 1.69, 95% CI = 1.01–2.80, *p* = 0.043) among women. It revealed that the effects of *ADCY9* gene polymorphism on HCC risk were dependent on age or gender. Nevertheless, there was no significant association of these five polymorphisms with the risk of HCC in the men group. After FDR correction, no remarkable linkages were shown in the subgroups, suggesting that the differences in age or gender may not affect the relationship of *ADCY9* gene polymorphism and the susceptibility of HCC.

**Table 4:** The association between *ADCY9* gene polymorphisms and hepatocellular carcinoma risk in subgroup of age.

**SN**	**Model**	**Age (>55)**				**FDR-*p***	**Age (≤55)**				**FDR-*p***
		**Without adjustment**		**With adjustment**			**Without adjustment**		**With adjustment**		
		**OR (95% CI)**	***p***	**OR (95% CI)**	***p***		**OR (95% CI)**	***p***	**OR (95% CI)**	***p***	
rs2531995	Homozygote	1 (0.47–2.12)	0.991	1.01 (0.47–2.15)	0.990	0.990	1.38 (0.8–2.38)	0.240	1.41 (0.82–2.44)	0.218	0.327
	Heterozygote	1.44 (0.96–2.17)	0.081	1.44 (0.95–2.17)	0.082	0.595	1.28 (0.87–1.91)	0.213	1.32 (0.88–1.96)	0.175	0.292
	Dominant	1.36 (0.92–2.01)	0.124	1.36 (0.92–2.02)	0.123	0.595	1.31 (0.91–1.89)	0.152	1.34 (0.92–1.95)	0.122	0.305
	Recessive	0.84 (0.4–1.75)	0.645	0.85 (0.41–1.78)	0.666	0.805	1.22 (0.74–2.01)	0.445	1.23 (0.74–2.04)	0.432	0.589
	Additive	1.18 (0.86–1.61)	0.305	1.18 (0.86–1.61)	0.297	0.861	1.2 (0.93–1.55)	0.165	1.22 (0.94–1.58)	0.139	0.298
	Allele			1.17 (0.86–1.59)	0.314	0.828			1.21 (0.93–1.58)	0.151	0.302
rs879620	Homozygote	0.86 (0.4–1.85)	0.695	0.86 (0.40–1.86)	0.700	0.812	1.71 (0.98–2.99)	0.058	1.75 (1.00–3.07)	0.051	0.191
	Heterozygote	1.21 (0.81–1.82)	0.356	1.21 (0.80–1.82)	0.359	0.744	1.38 (0.93–2.03)	0.111	1.43 (0.96–2.13)	0.078	0.234
	Dominant	1.15 (0.78–1.71)	0.124	1.15 (0.78–1.71)	0.479	0.695	1.45 (1.01–2.1)	**0.047**	1.50 (1.04–2.18)	**0.031**	0.133
	Recessive	0.78 (0.37–1.65)	0.516	0.78 (0.37–1.66)	0.522	0.688	1.46 (0.87–2.45)	0.154	1.46 (0.87–2.48)	0.155	0.291
	Additive	1.05 (0.77–1.44)	0.769	1.05 (0.77–1.44)	0.766	0.854	1.33 (1.02–1.72)	**0.034**	1.35 (1.04–1.76)	**0.026**	0.156
	Allele			1.05 (0.77–1.42)	0.776	0.833			1.35 (1.03–1.76)	**0.028**	0.140
rs2230742	Homozygote	0.44 (0.04–4.85)	0.499	0.42 (0.04–4.66)	0.476	0.727	0.36 (0.1–1.4)	0.141	0.34 (0.09–1.31)	0.117	0.319
	Heterozygote	0.83 (0.51–1.35)	0.453	0.83 (0.51–1.35)	0.449	0.723	0.6 (0.39–0.93)	**0.022**	0.57 (0.36–0.89)	**0.013**	0.098
	Dominant	0.81 (0.5–1.31)	0.394	0.81 (0.50–1.31)	0.387	0.660	0.58 (0.38–0.88)	**0.011**	0.54 (0.35–0.84)	**0.006**	0.090
	Recessive	0.45 (0.04–5.03)	0.519	0.43 (0.04–4.83)	0.495	0.684	0.41 (0.11–1.57)	0.193	0.39 (0.10–1.50)	0.170	0.300
	Additive	0.8 (0.51–1.27)	0.348	0.80 (0.51–1.26)	0.338	0.817	0.6 (0.41–0.88)	**0.009**	0.57 (0.39–0.84)	**0.004**	0.120
	Allele			0.81 (0.52–1.27)	0.358	0.799			0.59 (0.41–0.87)	**0.007**	0.070
rs2230741	Homozygote	1.9 (0.74–4.88)	0.183	1.87 (0.72–4.81)	0.196	0.812	0.53 (0.2–1.4)	0.199	0.54 (0.20–1.45)	0.221	0.316
	Heterozygote	1.03 (0.68–1.55)	0.893	1.03 (0.69–1.56)	0.875	0.906	1.39 (0.94–2.06)	0.095	1.44 (0.97–2.13)	0.068	0.227
	Dominant	1.11 (0.74–1.64)	0.618	1.11 (0.75–1.65)	0.611	0.770	1.25 (0.86–1.82)	0.236	1.30 (0.89–1.89)	0.179	0.283
	Recessive	1.88 (0.74–4.76)	0.184	1.84 (0.73–4.69)	0.199	0.721	0.47 (0.18–1.24)	0.127	0.47 (0.18–1.26)	0.136	0.314
	Additive	1.17 (0.84–1.62)	0.359	1.17 (0.84–1.62)	0.364	0.704	1.08 (0.78–1.48)	0.650	1.10 (0.80–1.52)	0.549	0.716
	Allele			1.16 (0.84–1.61)	0.365	0.662			1.08 (0.78–1.47)	0.651	0.751
rs2531992	Homozygote	0.14 (0.02–1.17)	0.070	0.13 (0.02–1.12)	0.063	0.914	1.67 (0.28–10.1)	0.577	1.66 (0.27–10.2)	0.583	0.700
	Heterozygote	0.76 (0.47–1.23)	0.266	0.75 (0.46–1.23)	0.255	0.822	0.93 (0.59–1.48)	0.771	0.92 (0.58–1.47)	0.733	0.814
	Dominant	0.68 (0.43–1.1)	0.114	0.68 (0.42–1.09)	0.106	0.615	0.96 (0.62–1.51)	0.868	0.95 (0.61–1.50)	0.828	0.887
	Recessive	0.15 (0.02–1.24)	0.078	0.14 (0.02–1.19)	0.071	0.686	1.69 (0.28–10.21)	0.567	1.69 (0.28–10.34)	0.571	0.714
	Additive	0.65 (0.42–1)	**0.049**	0.64 (0.42–0.99)	**0.044**	1.276	1.00 (0.66–1.51)	0.985	0.99 (0.65–1.50)	0.947	0.980
	Allele			0.64 (0.42–0.99)	**0.044**	1.276			1.00 (0.66–1.50)	0.985	0.985

*SNP, Single-nucleotide polymorphism; OR, Odds ratios; CI, Confidence intervals; FDR, False discovery rate*.

*Significant values are marked in bold*.

*“-” Means no data*.

*With adjustment means adjusted by gender*.

**Table 5:** The association between *ADCY9* gene polymorphisms and hepatocellular carcinoma risk in subgroup of gender.

**SNP**	**Model**	**Men**				**FDR-*p***	**Women**				**FDR-*p***
		**Without adjustment**		**With adjustment**			**Without adjustment**		**With adjustment**		
		**OR (95% CI)**	***p***	**OR (95% CI)**	***p***		**OR (95% CI)**	***p***	**OR (95% CI)**	***p***	
rs2531995	Homozygote	1.29 (0.78–2.15)	0.324	1.30 (0.78–2.17)	0.311	0.376	1.02 (0.43–2.38)	0.972	0.99 (0.42–2.34)	0.980	0.980
	Heterozygote	1.29 (0.94–1.76)	0.119	1.29 (0.94–1.76)	0.116	0.534	1.58 (0.83–3.03)	0.165	1.59 (0.83–3.05)	0.162	0.414
	Dominant	1.29 (0.95–1.73)	0.099	1.29 (0.96–1.74)	0.096	0.552	1.39 (0.77–2.51)	0.280	1.38 (0.76–2.50)	0.285	0.546
	Recessive	1.14 (0.7–1.85)	0.596	1.15 (0.71–1.86)	0.581	0.557	0.82 (0.37–1.83)	0.634	0.80 (0.36–1.80)	0.595	0.720
	Additive	1.19 (0.95–1.49)	0.138	1.19 (0.95–1.49)	0.131	0.430	1.11 (0.74–1.66)	0.620	1.10 (0.73–1.65)	0.641	0.737
	Allele			1.18 (0.95–1.48)	0.139	0.355			1.13 (0.73–1.74)	0.592	0.756
rs879620	Homozygote	1.39 (0.83–2.33)	0.217	1.39 (0.83–2.34)	0.210	0.403	1.12 (0.46–2.71)	0.803	1.09 (0.45–2.66)	0.854	0.893
	Heterozygote	1.2 (0.88–1.65)	0.246	1.21 (0.88–1.65)	0.245	0.352	1.7 (0.89–3.23)	0.108	1.72 (0.90–3.28)	0.101	0.290
	Dominant	1.24 (0.92–1.67)	0.165	1.24 (0.92–1.67)	0.163	0.341	1.51 (0.84–2.74)	0.171	1.51 (0.84–2.74)	0.171	0.393
	Recessive	1.26 (0.77–2.07)	0.354	1.27 (0.77–2.08)	0.344	0.377	0.88 (0.38–2.03)	0.764	0.86 (0.37–2.00)	0.722	0.791
	Additive	1.19 (0.95–1.49)	0.139	1.19 (0.95–1.49)	0.135	0.388	1.19 (0.78–1.79)	0.418	1.18 (0.78–1.79)	0.431	0.661
	Allele			1.18 (0.94–1.48)	0.143	0.329			1.21 (0.78–1.88)	0.388	0.686
rs2230742	Homozygote	0.54 (0.16–1.85)	0.325	0.54 (0.16–1.86)	0.326	0.375	-	-	-	-	-
	Heterozygote	0.75 (0.52–1.08)	0.118	0.75 (0.52–1.08)	0.120	0.460	0.48 (0.23–1.01)	0.053	0.48 (0.23–1.00)	0.050	0.192
	Dominant	0.73 (0.51–1.04)	0.084	0.73 (0.51–1.05)	0.086	0.659	0.43 (0.21–0.89)	**0.023**	0.43 (0.21–0.88)	**0.022**	0.169
	Recessive	0.57 (0.17–1.97)	0.376	0.57 (0.17–1.98)	0.378	0.395	-	-	-	-	-
	Additive	0.74 (0.54–1.03)	0.072	0.75 (0.54–1.03)	0.074	0.851	0.42 (0.21–0.84)	**0.013**	0.42 (0.21–0.83)	**0.012**	0.138
	Allele			0.74 (0.54–1.03)	0.071	1.633			0.43 (0.22–0.83)	**0.011**	0.253
rs2230741	Homozygote	0.87 (0.44–1.75)	0.704	0.87 (0.44–1.74)	0.696	0.593	2.71 (0.48–15.45)	0.261	2.65 (0.46–15.18)	0.274	0.573
	Heterozygote	1.09 (0.8–1.5)	0.587	1.09 (0.79–1.50)	0.595	0.547	1.84 (0.99–3.43)	0.055	1.85 (0.99–3.46)	0.052	0.171
	Dominant	1.06 (0.78–1.44)	0.700	1.06 (0.78–1.44)	0.710	0.583	1.90 (1.04–3.48)	**0.038**	1.91 (1.04–3.50)	**0.037**	0.213
	Recessive	0.85 (0.43–1.68)	0.634	0.84 (0.43–1.67)	0.626	0.554	2.15 (0.38–12.02)	0.386	2.11 (0.37–11.89)	0.398	0.654
	Additive	1.02 (0.79–1.31)	0.886	1.02 (0.79–1.31)	0.898	0.688	1.78 (1.04–3.05)	**0.037**	1.78 (1.04–3.06)	**0.037**	0.213
	Allele			1.02 (0.79–1.31)	0.886	0.703			1.69 (1.01–2.80)	**0.043**	0.198
rs2531992	Homozygote	0.49 (0.14–1.63)	0.243	0.48 (0.14–1.62)	0.239	0.366	-	-	-	-	-
	Heterozygote	0.87 (0.6–1.26)	0.451	0.87 (0.60–1.26)	0.451	0.451	-	-	-	-	-
	Dominant	0.83 (0.58–1.19)	0.311	0.83 (0.58–1.19)	0.310	0.396	0.78 (0.36–1.68)	0.522	0.78 (0.36–1.69)	0.534	0.768
	Recessive	0.5 (0.15–1.68)	0.261	0.50 (0.15–1.67)	0.258	0.349	-	-	-	-	-
	Additive	0.82 (0.59–1.13)	0.221	0.82 (0.59–1.13)	0.219	0.360	0.78 (0.36–1.68)	0.522	0.78 (0.36–1.69)	0.534	0.768
	Allele			0.81 (0.59–1.13)	0.213	0.377			0.80 (0.38–1.66)	0.543	0.735

*SNP, Single-nucleotide polymorphism; OR, Odds ratios; CI, Confidence intervals; FDR, False discovery rate*.

*Significant values are marked in bold*.

*“-” Means no data*.

*With adjustment means adjusted by age*.

### Haplotype Analysis of Polymorphisms in *ADCY9*

**Figure 1:**
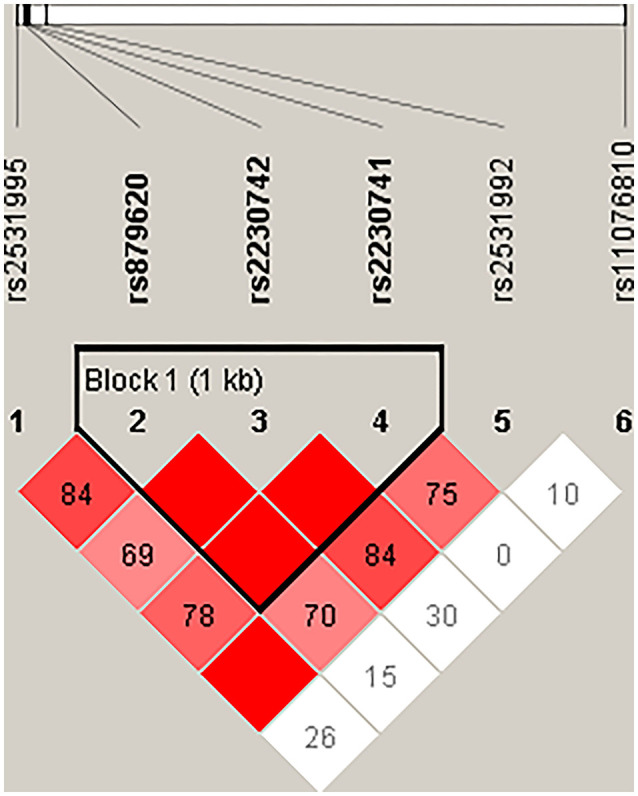
Haplotype block map for the SNPs of *ADCY9*. Block includes rs879620, rs2230742, and rs2230741. The LD between two SNPs is standardized D′.

Then, we performed the LD and haplotype analysis of these five polymorphisms to HCC risk. As shown in Figure 1, one block including rs879620, rs2230742, and rs2230741 was detected. In Table 6, the results indicated that the haplotype C-A-A (rs879620, rs2230742, and rs2230741) was associated with the decreased risk of HCC (adjusted OR = 0.67, 95% CI = 0.50–0.89, *p* = 0.007, FDR-*p* = 0.028).

**Table 6:** Associations between *ADC*Y9 haplotype frequencies and hepatocellular carcinoma risk.

**SNP**	**Haplotype**	**Freq-case**	**Freq-control**	**Without adjustment**		**With adjustment**		**FDR- *p***
				**OR (95% CI)**	***p***	**OR (95% CI)**	***p***	
rs879620|rs2230742|rs2230741	CGG	0.767	0.788	0.89 (0.71–1.11)	0.297	0.89 (0.71–1.11)	0.298	0.397
rs879620|rs2230742|rs2230741	CAA	0.103	0.146	0.67 (0.50–0.90)	**0.007**	0.67 (0.50–0.89)	**0.007**	**0.028**
rs879620|rs2230742|rs2230741	TGA	0.652	0.689	0.84 (0.69–1.03)	0.096	0.84 (0.69–1.03)	0.094	0.188
rs879620|rs2230742|rs2230741	CGA	0.684	0.669	1.07 (0.88–1.30)	0.513	1.07 (0.88–1.30)	0.507	0.507

*SNP, Single-nucleotide polymorphism; OR, Odds ratios; CI, Confidence intervals; FDR, False discovery rate*.

*Significant values are marked in bold*.

*With adjustment means adjusted by age and gender*.

## Discussion

In the present study, we focused on the Chinese Han population and found that the AA, AA-AG genotype, and the A allele of rs2230742 could decrease the risk of HCC. Moreover, rs2230742 was related to HCC risk in the subgroup of younger participants (age ≤ 55 years old) and women, indicating that subjects with A allele of rs2230742 are less likely to have HCC. After FDR analysis, rs2230742 was still significantly associated with lower risk of HCC in the allele model. It means that rs2230742 may be a potential protective factor for HCC and help to guide treatment, and rs2230742 possibly affects the susceptibility of HCC by associating with “SiPhy cons,” “DNAse,” and “Motifs changed.” However, there were no significant linkages between other polymorphisms and HCC susceptibility after FDR correction. Besides that, C_rs879620_A_rs2230742_A_rs2230741_ haplotype could protect individuals from HCC (OR = 0.67, 95% CI = 0.50–0.89, *p* = 0.007, FDR*-p* = 0.028). These results suggested that *ADCY9* gene polymorphisms might be involved in the susceptibility of HCC in the Chinese Han population.

*ADCY9* is a widely distributed adenylyl cyclase, which catalyzes the formation of cyclic AMP from ATP. Human *ADCY9* is stimulated by beta-adrenergic receptor activation but is insensitive to forskolin, calcium, and somatostatin (10). Defects in *ADCY9* gene can lead to immune-mediated diseases (14). Nevertheless, the role of *ADCY9* in tumorigenesis is still not clear. *ADCY9* expression was found to be significantly different in endometrial cancer when compared to the controls, which might be involved in the pathogenesis of this cancer (19). In addition, *ADCY9* is a known target of microribonucleic acids-−142-3p, which is associated with the invasiveness of breast cancer cells (20). Previous studies also reported that the SNPs in *ADCY9* were associated with stroke, malaria, medicine responses, and cancer (9, 21–23). Rs2230739 of *ADCY9* was involved in various pathways and processes, which might contribute to the susceptibility of pancreatic cancer (24). However, no significant associations were reported between *ADCY9* and diseases. Our study firstly demonstrated that *ADCY9* gene polymorphisms were associated with HCC risk, especially rs2230742 significantly altered the susceptibility of HCC, and it confirmed that *ADCY9* was involved in cancer development in previous studies (9). However, further studies on the molecular function of *ADCY9* SNPs should be performed to decipher its role in HCC.

Age and gender are considered as risk factors in the development of cancer, including HCC. The incidence of HCC increases with age, and it is the highest in individuals around the age of 70 (25). It also provided that males have higher liver cancer rates than females (26). Then, we did stratification analysis by age and gender. We found that *ADCY9* gene polymorphisms altered HCC susceptibility in the subgroups except for men. Among them, rs2230742 significantly decreased HCC risk in the subgroup of women and individuals younger than 55 years old. Rs2531992 was also associated with a decreased risk of HCC for the elderly people (age > 55). Nevertheless, rs879620 and rs2230741 were associated with higher risk of HCC for the individuals aged 55 or younger and women, respectively. It suggests that the influence of *ADCY9* gene polymorphisms on HCC risk is age- and gender-dependent, which may be helpful for the individual treatment of HCC in the Chinese Han population. After FDR correction, no significant associations were observed in the subgroups. It suggests that some positive findings might be caused by false positives. In the future, more studies are required to verify the influences of age and gender on the association of *ADCY9* gene polymorphisms with HCC risk.

We further conducted haplotype analysis in order to demonstrate whether the interaction of these five SNPs has effect on HCC risk. Analysis of the results indicates that the *ADCY9* haplotype CAA (rs879620, rs2230742, and rs2230741) is associated with a decreased risk of HCC, which may suggest that these SNPs work together. There is also a probability that this haplotype is a genetic marker for a rare mutation among the Chinese Han population.

Our study also had some limitations. First, this is a single central study, so selection bias is inevitable. Then, we did not analyze the influence of lifestyle factors and other risk factors because of lacking related information. Hence, further studies are necessary to confirm the association between *ADCY9* gene polymorphisms and HCC risk.

## Conclusion

To sum up, the present study suggests that the *ADCY9* gene polymorphisms (rs2531995 and rs2230742) are associated with HCC susceptibility in the Chinese Han population and may be involved in tumor development. Additionally, the relationships of *ADCY9* gene polymorphisms and HCC susceptibility are age- and gender-dependent; it may guide us to individual treatment. Further functional studies and large population with more races are needed to confirm the influence of *ADCY9* variants on HCC risk.

## Data Availability Statement

All datasets generated for this study are included in the article/Supplementary Material.

## Ethics Statement

This study was reviewed and approved by the Ethics Committee of Shaanxi University of Chinese Medicine (application number: SQ2020024), and written informed consents were collected from all participates. The research protocol was completed according to the Declaration of Helsinki.

## Author Contributions

XC and CJ designed and supervised this study. YJ, XF, GW, and XW mainly performed this study. HS analyzed the data. FZ wrote this manuscript. All authors contributed to the article and approved the submitted version.

## Conflict of Interest

The authors declare that the research was conducted in the absence of any commercial or financial relationships that could be construed as a potential conflict of interest.
