# Supplementary material for: A Case–Control Study of ADCY9 Gene Polymorphisms and the Risk of Hepatocellular Carcinoma in the Chinese Han Population
Source: Front Oncol. 2020 Aug 25;10:1450. doi: 10.3389/fonc.2020.01450 (PMC7477943; doi:10.3389/fonc.2020.01450)
Supplement: Supplementary file 1 [file Table_1.DOCX]

**Supplemental Table 1** Primers used in the study

| **SNP** | **1st-PCRP** | **2nd-PCRP** | **UEP_DIR** | **UEP_SEQ** |
| --- | --- | --- | --- | --- |
| rs2531995 | ACGTTGGATGTGAGCCTTCCTAAACAGCCA | ACGTTGGATGTGAGGTATGCATCGGAGCAG | F | GTGGCCCAGAGAGAA |
| rs879620 | ACGTTGGATGTGGCGCTTGGAAAGCACAAC | ACGTTGGATGTTCAAAGAGTGTGTGAGGCG | R | agagCTGTTTGTCGAAACACA |
| rs2230742 | ACGTTGGATGTGAAGGTGTCCCAGACCTAC | ACGTTGGATGAGAACTCGCTGAAGTTGACG | F | gaagcTCACCCCTCCGCTGTC |
| rs2230741 | ACGTTGGATGGAAAGAAGACGAGAACCACC | ACGTTGGATGTCTTTGCTCTCCAGTTCCGA | R | AGAGGAACCCGTGCAATAG |
| rs2531992 | ACGTTGGATGTCGTTGGGTTAGGAGTTGAC | ACGTTGGATGGGATGTTAGACCTTGGTGAC | R | actaCTAAAGGGGACTGTTCT |
